# Tuberculosis mortality: patient characteristics and causes

**DOI:** 10.1186/1471-2334-14-5

**Published:** 2014-01-03

**Authors:** Chou-Han Lin, Chou-Jui Lin, Yao-Wen Kuo, Jann-Yuan Wang, Chia-Lin Hsu, Jong-Min Chen, Wern-Cherng Cheng, Li-Na Lee

**Affiliations:** 1 Department of Internal Medicine, Far Eastern Memorial Hospital: No.21, Sec. 2, Nanya S. Rd., Banciao Dist., New Taipei City, Taiwan; 2 Department of Internal Medicine, Tao-Yuan General Hospital, No.1492, Chung-Shan Road, Taoyuan City, Taoyuan County, Taiwan; 3 Department of Internal Medicine, National Taiwan University Hospital, No.7, Chung-Shan South Road, Taipei, Taiwan; 4 Department of Laboratory Medicine, National Taiwan University College of Medicine and Hospital, No.7, Chung-Shan South Road, Taipei 10002, Taiwan

**Keywords:** Tuberculosis, Mortality, Risk factor

## Abstract

**Background:**

In the antibiotic era, tuberculosis (TB) still causes a substantial number of mortalities. We aimed to identify the causes and risks of death among TB patients.

**Methods:**

Medical records of mortality cases of culture-proven TB diagnosed during 2003–2007 were reviewed. All TB deaths were classified into 2 groups (TB-related and non-TB-related), based on the underlying cause of death.

**Results:**

During the study period, 2016 cases (male: 71.1%) of culture-proven TB were identified. The mean age was 59.3 (range: 0.3–96) years. The overall mortality rate was 12.3% (249 cases) and the mean age at death was 74 years; 17.3% (43 cases) of all TB deaths were TB-related. Most of the TB-related deaths occurred early (median survival: 20 days), and the patient died of septic shock. Malignancy, liver cirrhosis, renal failure, and miliary and pneumonic radiographic patterns were all independent predictors for all TB deaths. Cavitary, miliary and pneumonic radiographic patterns were all significant predictive factors for TB-related death. Extrapulmonary involvement and liver cirrhosis were also factors contributing to TB-related death.

**Conclusions:**

The majority of TB deaths were ascribed to non-TB-related causes. Managing TB as well as underlying comorbidities in a multidisciplinary approach is essential to improve the outcome of patients in an aging population. However, the clinical manifestations of patients with TB-related death vary; many progressed to fulminant septic shock requiring timely recognition with prompt treatment to prevent early death.

## Background

Tuberculosis (TB) remains a serious public health issue worldwide. Even in the era of effective chemotherapy, TB still accounts for a substantial number of deaths annually. Early diagnosis is challenging, even in areas with abundant medical resources [1]. In 2012, there were an estimated 12 million TB cases globally, including 8.6 million new cases, and 1.3 million fatal cases [2]. The global case-fatality rates are reported to be between 7% and 35% [3], and risk factors for death may include non-infective comorbidities, human immunodeficiency virus (HIV) infection and multidrug-resistant TB (MDRTB) [4]. Since the World Health Organization (WHO) defined TB deaths as the number of TB patients dying during treatment, irrespective of cause [5], most studies have used all-cause mortality as a surrogate marker of mortality attributable to TB [3,6-8]. Nevertheless, knowing the actual underlying cause of death, especially whether it was TB-related or not, is valuable in monitoring TB control and may help in identifying effective interventions [9,10].

Some studies have investigated the actual causes of death among TB patients [10-17], and most relied on vital statistics registration or death certificates [10,11,13-15,17]. However, they may not completely reflect the actual causes of death because of reporting bias due to inaccurate certificates in the registration system and the imprecise design in large population-based surveys [18-21]. Moreover, these studies were conducted in areas with a high prevalence of either HIV infection or MDRTB [3,10-13]. These data may not be applicable to the rest of the world, and to countries such as Taiwan with an intermediate TB burden (incidence: 58/100,000; mortality: 3.2/100,000), and a low prevalence of MDRTB (1%) and HIV infection (0.7%) among newly-diagnosed TB cases [22]. Hence, we conducted this study to investigate the causes and risk factors for death among TB patients in Taiwan.

## Methods

This study was conducted in a 2500-bed university-affiliated hospital in northern Taiwan, and was approved by the ethics committee of National Taiwan University Hospital. In the approved protocol, the requirement for informed consent was waived due to the retrospective nature of the study. We searched mycobacterial laboratory and histology databases from 2003 through 2007 for all patients with newly diagnosed, culture-proven TB. We reviewed their medical records and obtained the following data: demographic characteristics, comorbidities, indications for seeking medical help, symptoms, radiological appearance, bacteriological investigation, laboratory findings, HIV serology, length of hospital stay and outcome. Along with HIV serostatus, all patients were followed for acquired immunodeficiency syndrome (AIDS)-defined illness until the end of the study. According to the WHO definition, TB death was defined as all-cause mortality before completing anti-TB treatment. All of our patients were followed until completely treated, death, or until December 31, 2010 (end of the study).

All TB deaths were classified into 2 groups according to the underlying cause of death – TB-related or non-TB-related, rather than the mode of death, such as respiratory failure or septic shock. In order to find the risk factors contributing to TB-related death without a confounding element, the defined TB-related death had to fulfill the following 3 criteria: 1) microbiological or pathologic evidence of sole TB infection without other pathogens cultured from sterile body fluid or tissue aseptically collected; 2) agreement between the reviewing physician and the underlying cause of death recorded on the death certificate or medical records from the primary care physician; and 3) no other cause that was equally likely to result in mortality. Otherwise, the mortality was classified as non-TB-related death, and the underlying cause of death was further determined. The mode of death for TB-related death was also recorded and defined as: 1) “respiratory failure” that preceded shock (septic or non-septic), with acute lung injury (ALI) or acute respiratory distress syndrome (ARDS). ALI or ARDS was diagnosed based on the consensus of the American-European Conference [23]; 2) “septic shock” preceding respiratory failure with or without ALI/ARDS. Patients with septic shock were required to have systemic inflammatory response syndrome, documented or suspected infection, and persistent hypotension despite fluid resuscitation [24]; and 3) “others”.

Survival time was defined as the interval between the day the index culture was plated and the time of death. An interval longer than 14 days between ordering the index TB culture and commencing anti-TB treatment was defined as a delay in treatment.^2^ Multidrug resistance (MDR) was defined as resistance against at least isoniazid and rifampicin. Chest radiographs of patients with TB-related death were categorized into miliary, cavitary or pneumonic patterns. A miliary pattern was defined as diffuse millet-sized nodules, a cavitary pattern was defined as radiographic opacity with an internal area of lucency, and a pneumonic pattern was defined as consolidation or infiltrates resembling bacterial pneumonia. When the chest radiograph pattern did not meet the above definitions, the film was classified as “others”.

For each mortality case, 2 age- (within 2 years), sex- and site of TB- (pulmonary or extrapulmonary) matched controls were selected. If sufficient controls could not be obtained, the acceptable age range was widened to 5 years. Controls were randomly selected from the baseline cohort of culture-proven TB patients diagnosed between 2003 and 2007 who completed a standard course of anti-TB treatment.

### Statistical analysis

Data were expressed as percentage of the group for categorical variables and mean ± standard deviation (SD) for continuous variables, and were compared using Pearson’s X^2^ test or Fisher’s exact test and the independent-samples *t* test, respectively. For cases of TB-related death and non-TB-related death, comparison with the control group was performed. Survival curves were generated using the Kaplan-Meier method for all TB patients. Cox’s proportional hazards analysis for all variables (age, sex, sputum acid-fast smear, extrapulmonary involvement, malignancy, diabetes mellitus, liver cirrhosis, renal failure, HIV/AIDS, MDRTB, radiographic patterns, and delayed treatment) was employed to identify independent prognostic factors for all TB deaths in the initial model. In the subpopulation analysis of all TB deaths, TB-related death was compared with the control group. Age, sex and site of TB were not specifically matched with controls in this second model. Statistical analysis was performed with SPSS software (version 13.0 SPSS, Inc., Chicago, IL, USA).

## Results

### All-cause mortality among TB patients

**Figure 1:**
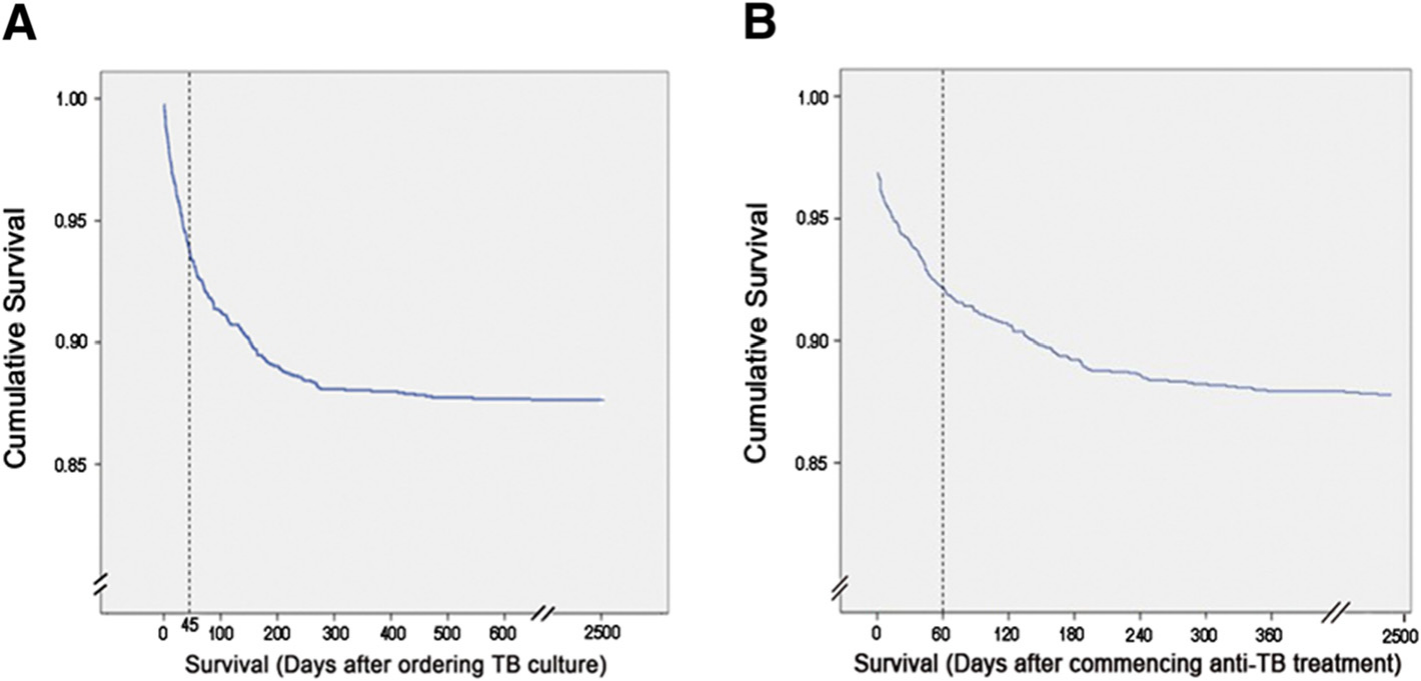
**Kaplan-Meier survival curve of all TB patients.** Panel **A** and **B** shows the survival curve after ordering TB culture and commencing anti-TB treatment, respectively.

A total of 2016 patients (male: 71.1%) with culture-proven TB were identified from 2003 to 2007. Their mean age was 59.3 (range: 0.3–96) years. Of the 2016 patients, 249 (12.4%) (male: 72.3%) died before completing anti-TB treatment. Their mean age was 74 (range: 12–95) years. Patients who were among the first half of all TB deaths succumbed within 45 days (Figure 1A). Sixty (3%) patients were diagnosed postmortem without receiving anti-TB treatment, and 98 (4.9%) patients died during the initial 2-month intensive phase of anti-TB treatment (Figure 1B). TB was the underlying cause of death of 43 (2.1%) patients (male: 62.8%; mean age: 75). For the remaining 206 patients (male: 74.3%; mean age: 74) who died of non-TB-related causes, malignancy (47.6%) was the leading cause (Table 1). The underlying cause of death of 7 patients was classified as unknown because of the disagreement between the reviewing physician and the primary care physician. TB was diagnosed postmortem in 37.2% of the TB-related deaths and 21.3% of the non-TB-related deaths. Median survival for patients who died of TB and non-TB-related causes was 20 (range: 1–423) and 55 (range: 1–704) days, respectively (*p* < 0.001 by log-rank test).

**Table 1:** Underlying cause of death and median survival days among 249 culture-proven tuberculosis (TB) mortality cases

**Underlying cause of death**	**Number (%)**	**Median survival days (range)**
TB	43 (17.2)	20 (1–423)
Non-TB	206 (82.7)	55 (1–704)
Malignancy	98 (39.3)	57 (1–704)
Bacterial infection	32 (12.8)	55 (1–206)
CVA	20 (8)	62 (2–544)
Hepatic failure	12 (4.8)	75 (9–365)
Renal failure	10 (4)	57 (4–136)
Cardiovascular disease	9 (3.6)	50 (1–270)
Autoimmune	6 (2.4)	45 (6–269)
COPD with respiratory failure	5 (2)	30 (1–88)
HIV/AIDS	2 (0.8)	31 (17–45)
Others*	12 (4.8)	87 (7–472)
Total	249	45 (1–704)

COPD: chronic obstructive pulmonary disease; CVA: cerebral vascular disease; HIV: human immunodeficiency virus; AIDS: acquired immunodeficiency syndrome.

*Including 4, 1 and 7 patients that died of accidents, primary pulmonary hypertension, and unknown causes, respectively.

### Characteristics of all TB deaths: TB-related death vs. non-TB-related death

There were 498 matched controls. Of the comorbidities, malignancy was more common in non-TB-related deaths than in the controls. In addition, both TB- and non-TB-related deaths were associated with significantly more liver cirrhosis and renal failure (Table 2).

**Table 2:** Characteristics of those with TB-related death and non-TB-related death and controls

	**TB-related death (n = 43)**	**Non-TB-related death (n = 206)**	**Control (n = 498)**	***p *** **value**
Age (years): mean	75	74	72	0.55
Male (%)	27 (62.8)	153 (74.3)	362 (72.7)	0.31
AFB smear-positive sputum (%)	12 (27.9)	49 (19.4)	99 (19.9)	0.43
Extrapulmonary involvement	25 (58.1)	41 (19.9)	132 (26.5)	<0.001
TB pleurisy or peritonitis	10 (23.3)	28 (13.6)	54 (10.8)	0.05
TB lymphadenitis	0	1 (0.5)	6 (1.2)	0.54
Urogenital TB	2 (4.7)	2 (1)	4 (0.8)	0.06
Disseminated TB	13 (30.2)	5 (2.4)	34 (6.8)	<0.001
with meningitis	3	0	6	
without meningitis	10	5	28	
Gastrointestinal TB	0	3 (1.5)	2 (0.4)	0.25
Musculoskeletal TB	0	2 (1)	26 (5.2)	0.01
TB meningitis	0	0	6 (1.2)	0.22
Hemoglobin (g/dl): mean	10.6	11.7	11.4	0.001
Comorbidity (%)	23 (53.5)	162 (78.6)	184 (36.9)	<0.001
Malignancy	9 (20.9)	114 (55.3)	61 (12.2)	<0.001
Diabetes mellitus	10 (23.3)	55 (26.7)	117 (23.5)	0.66
Liver cirrhosis	6 (14)	25 (12.1)	7 (1.4)	<0.001
Renal failure	4 (9.3)	24 (11.7)	15 (3.0)	<0.001
HIV/AIDS (%)	2 (4.7)	2 (1.0)	9 (1.8)	0.24
MDRTB (%)	0	5 (2.4)	13 (2.6)	0.57
Radiographic pattern				
Pneumonic patch	31 (72.1)	142 (68.9)	210 (42.2)	<0.001
Miliary shadow	4 (9.3)	6 (2.9)	24 (4.8)	0.17
Cavity	4 (9.3)	9 (4.4)	58 (11.6)	0.01
Other pattern	0	36 (17.5)	184 (36.9)	<0.001
No parenchymal lesion	4(9.3)	13 (6.3)	22 (4.4)	0.28
Delay in treatment >14 days	23 (53.5)	109 (52.9)	228 (45.8)	0.18

AFB: acid-fast bacilli; HIV: human immunodeficiency virus; AIDS: acquired immunodeficiency syndrome; MDR: multidrug resistant; TB: tuberculosis.

Data were percentages unless otherwise described.

*p*-value for comparisons between mortality cases and survivors.

### Clinical profiles of patients with TB-related death

Only 8 cases were diagnosed as TB initially. Pneumonia was the most common tentative diagnosis, and 32.6% of patients did not present with pulmonary symptoms (Table 3). Of the 43 mortality cases, and apart from the stool specimen, all of the extrapulmonary TB was diagnosed by culture from an aseptic site (blood and urine) or invasive procedures (thoracentesis, paracentesis, bone marrow biopsy, lumbar puncture and arthrocentesis). Over 80% of patients presented with a pneumonic pattern, and only 9% showed miliary TB. The median time from arriving at the hospital to performing mycobacterial culture was 3 days (interquartile range: 0–17). Sixteen (37.2%) cases were diagnosed postmortem, without ever receiving anti-TB medication; 14 (32.5%) experienced a rapid fatal course and died in less than 14 days. Among the 27 patients diagnosed ante-mortem, the median interval between the initial visit and starting anti-TB treatment was 12 days (interquartile range: 1–27), and 16 (59.3%) of these patients had received anti-TB treatment for ≥14 days before death. The median interval from treatment initiation to death was 23 days (interquartile range: 8–48). Twenty-three of the 43 TB-related mortality cases were admitted to the intensive care unit for mechanical ventilation or septic shock management (vasopressor, fluid resuscitation and continuous hemodynamic monitoring). The final mode of death was septic shock in 20 patients (46.5%), respiratory failure in 18 (41.9%), and TB-related cachexia in the remaining 5 (11.6%), including massive gastrointestinal bleeding in 2, suffocation in 2, and sudden cardiac arrest in one (Table 4).

**Table 3:** Clinical presentation of 43 patients with TB-related death

	**Number (%)**
Initial tentative diagnosis	
TB	8 (18.6)
Non-TB infection	26 (60.5)
Pneumonia	17
Urosepsis	2
Septic shock	2
Neutropenic fever	1
Spontaneous bacterial peritonitis	1
Meningitis, nature undetermined	1
Cellulitis	1
Fever of unknown origin	1
Noninfectious diagnosis	9 (20.9)
Acute myocardial infarction	2
Cancer progression	2
COPD with acute exacerbation	1
Chronic diarrhea	1
Hydrocephalus	1
Choledocholithiasis	1
Nephrotic syndrome	1

COPD: chronic obstructive pulmonary disease.

**Table 4:** Laboratory findings, thoracic radiographic presentations and clinical course of 43 patients with TB-related death

	**Number (%)**
Radiographic pattern	
Pneumonia	31 (72)
Miliary shadows	4 (9.3)
Cavitary lesion	4 (9.3)
No parenchymal lesion	4 (9.3)
Platelet (10^9^/L) <140	16 (37.2)
Albumin (g/dL) <3.5	34 (79)
White blood cell (10^9^/L) ≥9	13 (30.2)
Hemoglobin (g/dL) <10	15 (34.9)
No. (%) of samples that grew *M. tuberculosis*	
Sputum	30(69.8)
Pleural effusion	12(27.9)
Blood	6(14)
Ascites	4(9.3)
Cerebrospinal fluid	3(7)
Bone marrow	1(2.3)
Urine	1(2.3)
Stool	1(2.3)
Joint pus	1(2.3)
Delay from first visit to ordering TB culture >3 days	17 (39.5)
Mortality within 14 days	14 (32.5)
Ante-mortem diagnosis	27 (62.8)
Postmortem diagnosis	16 (37.2)

### Risk factors for all TB deaths and TB-related death

In a Cox regression model in which all TB deaths were compared to the controls, malignancy, liver cirrhosis, and renal failure were found to be independent prognostic predictors. Miliary and pneumonic radiographic patterns were also found to be associated with mortality. In the second model specifically regarding TB-related death, extrapulmonary involvement, liver cirrhosis, and miliary and pneumonic radiographic patterns remained as independent factors affecting survival (Table 5).

**Table 5:** Factors contributing to all TB deaths and TB-related death using the Cox-proportional hazard model

	**Variable**	**HR**	**95% CI**	***p*-value**
All TB deaths	Malignancy	3.91	3.03-5.04	<0.001
	Liver cirrhosis	3.18	2.17-4.68	<0.001
	Renal failure	2.81	1.89-4.19	<0.001
	Miliary pattern on CXR	2.25	1.15-4.40	0.02
	Pneumonic pattern on CXR	2.26	1.70-3.02	<0.001
TB-related death	Extrapulmonary involvement	4.80	2.57-8.97	<0.001
	Pneumonic pattern on CXR	8.66	2.99-25.12	<0.001
	Milary pattern on CXR	6.08	1.52-24.38	0.01
	Cavitary pattern on CXR	5.24	1.29-21.18	0.02
	Liver cirrhosis	5.07	2.07-12.40	<0.001

CXR: chest X-ray.

## Discussion

Our study has confirmed that there are a substantial number of deaths associated with TB, even in the era of effective anti-TB medication and advanced mycobacteriology laboratories. The all-cause mortality rate of TB patients was 12.4%, and this was mainly due to non-TB-related causes (82.7%). Malignancy, liver cirrhosis, renal failure, and miliary and pneumonic radiographic patterns predicted mortality in all TB deaths. Patients who died of TB progressed rapidly and 37.2% was not diagnosed ante-mortem.

Our study showed a TB-related mortality rate of 2.1%, which was comparable to studies in Canada [15,25] and in metropolitan area in China [10]. A study in the USA reported a TB-related case fatality rate of 0.1%, but more severe forms, such as those with extrapulmonary involvement or a poor performance status, were excluded from the study [17]. TB-related mortality rates in some regions of the world were higher than those in our study. Those are regions with more indigenous groups, poor access to health care or a higher MDRTB percentage, such as South Africa (7%) [10], Australia (8.7%) [14] and Russia (5.9-6.8%) [12,13]. We also found that 82.7% of the deaths were due to non-TB-related causes. This is consistent with previous studies that have shown aging and underlying comorbidities as risk factors for TB deaths in developed or developing countries [11,17]. This percentage was 98% in North America [17], 86% in the Netherlands [7], 75% in Russia [13] and 50.5% in China [11]. Interventions and team care to treat TB and coexisting diseases are needed to decrease overall mortality [17].

Those who died of TB had a short median survival (20 days). The median time from visit to ordering TB culture, however, was only 3 days, suggesting that physicians in our hospital were alert to TB. Yet more than one-third of those who died of TB did not receive anti-TB treatment before their death, probably because: 1) the patients often had negative sputum smears for acid-fast bacilli; 2) most patients whose diagnosis of TB was made postmortem (14/16, or 88%) had a rapidly fatal course (<14 days), and succumbed before culture results became available; 3) patients in the fatality group often showed a pneumonic pattern on chest radiograph, and were treated as having bacterial pneumonia; 4) they were often older patients with underlying comorbidity and a high risk of developing adverse drug effects, so physicians may not start empirical anti-TB treatment even when TB is highly suspected.

On the other hand, even among patients whose TB was diagnosed ante-mortem, the median time from treatment initiation to death (23 days) was short. This finding was similar to that of previous reports, in which many patients died of TB within a short period of time, ranging from 1 week to 3 months after starting treatment [7,9,13-15]. It was speculated that these patients were too ill on arrival and their outcomes could not be reversed even after treatment [16]. The initial tentative diagnosis of our fatal TB patients was bacterial pneumonia in 39.5%, and sepsis or other severe infections (e.g. peritonitis, meningitis, etc.) in 20%, suggesting that TB should be included in differential diagnoses in patients presenting with severe pneumonia, sepsis or other severe infections such as meningitis (Table 3). Our study, similar to a previous report, failed to demonstrate that delayed treatment was an important factor for mortality [3]. This finding does not minimize the importance of a prompt diagnosis and treatment of TB; rather the negative association is probably related to the retrospective design and definition of delayed treatment. Our observation that 40.7% of those who were diagnosed ante-mortem had received anti-TB treatment for less than 14 days before death suggested that some of the mortality may have been reversible if anti-TB therapy were started earlier.

Several comorbidities that are risk factors for all-cause mortality during anti-TB treatment have been noted. However the results were not consistent, probably due to the heterogeneous nature of the studied population. In a review by Waitt [4], comorbidities were risk factors for TB deaths in areas with high TB incidence and HIV prevalence, rather than in areas with low TB incidence and HIV prevalence. In our study, with TB and non-TB-related death defined separately, only liver cirrhosis was found to be a risk factor for TB-related death. Two case–control studies in the literature also reported that liver disease or hyperbilirubinemia was an independent risk factor for TB-related mortality [12,14]. The association of liver disease and TB-related mortality has seldom been reported, and this could be due to the lack of availability of diagnostic facilities for liver disease in resource-poor regions. This also indicates that physicians in resource-limited areas should be proactive in treating TB patients with physical signs of liver disease.

Previously reported TB-related critical conditions were mainly miliary TB with respiratory failure and ARDS [26,27]. In our observation, most (17/43, 39.5%) of the mortality cases presented with a pneumonic radiographic pattern and the patients died of septic shock rather than respiratory failure in a catastrophic course. Only 4 patients (9.3%) presented with miliary TB. Disseminated TB is difficult to diagnose if miliary lesions are not present on the radiograph [28]. TB with either a miliary or diffuse pneumonic pattern can progress to septic shock with multiple organ failure. This is termed *sepsis tuberculosa gravissima*, a condition that was described well before 1951 [29], but seemed to be left unnoticed after the advent of effective chemotherapy. However, in TB-non-endemic areas, it has been reported in non-HIV patients since late 1990 [30-32]. These patients often presented with bilateral diffuse alveolar and interstitial infiltrates, septic shock and hypoxemic respiratory failure. The refractory septic shock often led to multi-organ failure and death in hours or days. Therefore, to prevent these deaths, a high index of suspicion for TB needs to be maintained and prompt treatment should be initiated in pneumonic patients at risk of TB. The presence of diffuse pneumonic infiltrates and the high yield of sputum culture for *M. tuberculosis* (69.8% in our study) also suggest that sputum and bronchoalveolar lavage fluid PCR for *M. tuberculosis* may help an early diagnosis. However, the low positive rate of acid-fast stain smear in our study suggested that we may need to consider other rapid adjunctive tests, such as a urine lipoarabinomannan test for the early diagnosis of TB or extrapulmonary TB in resource-limited areas [33].

Our study has some limitations. First, we did not have autopsy reports, which are often considered the “gold standard” for underlying cause of death. However, misclassification bias should have been minimized by the strict criteria defining TB-related death. Second, co-infection with other bacteria was classified as non-TB-related death, according to the inclusion criterion. This may have resulted in an underestimation of the numbers of TB-related deaths. However, the strict criteria were used to prevent confounding while analyzing TB-related death. Third, our study was done retrospectively in an inner-city hospital, and information regarding many socio-cultural factors, such as homelessness, drug or alcohol addiction, HIV infection, etc., may not have been available. Thus our results may not be applicable to areas where these pre-hospital factors are important. Finally, gathering information on more detailed clinical parameters, such as central venous pressure or central venous oxygen saturation, was not done due to the retrospective nature of the study design. Further prospective clinical studies are needed for a better understanding of TB deaths.

## Conclusion

In the era of effective chemotherapy against TB, we found that a significant proportion of mortality among aging TB patients was due to underlying comorbidities. Liver cirrhosis was an important predictor for TB-related death. The majority of these patients presented as bacterial pneumonia and died rapidly due to septic shock. Our findings could help establish a better understanding of cause-specific TB mortality and help identify TB patients at risk.

## Competing interests

All of the authors declare no financial, professional, or otherwise personal interest of any nature or kind in any related product, service, and/or company.

## Authors’ contributions

LCH and LLN conceived and designed the study. LCH drafted the manuscript and all authors contributed substantially to its revision. LCJ and KWY were involved in the clinical data collection and gave critical review. WJY provided statistical advice. HCL, CJM, and CWC supervised the laboratory data collection and organized the database. All authors read and approved the final manuscript.

## Pre-publication history

The pre-publication history for this paper can be accessed here:

http://www.biomedcentral.com/1471-2334/14/5/prepub
